# Deepening trochleoplasty may dramatically increase retropatellar contact pressures- a pilot study establishing a finite element model

**DOI:** 10.1186/s40634-022-00512-9

**Published:** 2022-08-02

**Authors:** Dominik Kaiser, Tobias Götschi, Elias Bachmann, Jess G. Snedeker, Philippe M. Tscholl, Sandro F. Fucentese

**Affiliations:** 1 Department of Orthopedics, Balgrist University Hospital, University of Zurich, Forchstrasse 340, 8008 Zurich, Switzerland; 2 Department of Orthopedics, Biomechanical Research Laboratory, Balgrist Campus, University of Zurich, Zurich, Switzerland

## Background

The causes of patellofemoral instability are multifactorial and potentially complex [7, 12]. Multiple anatomical risk factors are known includingtrochlear dysplasia, an increased tibial tubercle- trochlear groove (TT-TG) distance and patella alta [10].

Recurrent patellar dislocations are strongly associated with cartilage lesions and onset of early patellofemoral osteoarthritis [3, 30]. Additionally, trochlear dysplasia may be an independent risk factor for early patellofemoral osteoarthritis even in the absence of dislocations [22].

The surgical goal in these patients is to stabilize the patella to reduce the number of dislocations potentially reducing the onset and evolution of patellofemoral cartilage degeneration.

In cases of severe trochlear dysplasia a widely used surgical approach to address the underlying pathology is trochleoplasty. Among the various surgical techniques described to address trochlear dysplasia through trochleoplasty [5, 7, 11, 34, 35], we perform sulcus deepening as described by Bereiter and Gautier [5], which may be one of the most commonly performed sulcus deepening procedures.

While there is literature describing surgical technique, predictors of clinical outcome as well as promising short- to mid-term clinical results [31, 34], to date there is little published knowledge on how the imposed geometric changes to the articulating surfaces by trochleoplasty may affect retropatellar cartilage stress.

The purpose of this study is to establish a finite element (FE) model to examine the retropatellar pressure distribution of a trochleodysplastic knee before and after simulated surgery as compared to that of a typical healthy knee without trochlear dysplasia. We will investigate how and to what extent the retropatellar pressure is affected and discuss whether this could potentially explain known risks of trochleoplasty, including postoperative degenerative changes and anterior knee pain [34, 39].

Our hypothesis is (a) that retropatellar pressure is higher in a trochleodysplastic knee compared to a healthy knee and that (b) surgical alteration of joint congruency will further increase retropatellar pressure.

## Methods

### Study material

MR image data of two female knees were selected as representative of their cohort (severe trochlear dysplasia and symptomatic; healthy and asymptomatic). The first was the right knee of a 20-year-old female (176 cm, 60 kg, BMI 19.37 kg/m^2^) that had suffered multiple non-traumatic patellar dislocations and was diagnosed with a severe type D trochlear dysplasia. The conventional radiographs showed a crossing sign, a supratrochlear spur, a double contour and a Caton-Deschamps Index of 1.2, the MRI showed a TT-TG distance of 17 mm, a patellar tilt of 18° and a bisect offset of 76%. As a healthy control a second model was established for an asymptomatic knee of a 24-year-old female (164 cm, 54 kg, BMI 20.08 kg/m^2^) with no medical history of knee pathology and an MRI without pathological findings, patellar tilt was 12°, bisect offset was 48.5%. Informed consent was obtained from both participants. Ethical approval to use the clinical data for research was obtained at the local ethics committee (KEK-ZH-Nr. 2014–0332).

### MR assessment

MR images were acquired with a sagittal high-resolution isotropic 3D PD SPACE sequence. A voxel size of 0.7 × 0.7 × 0.7mm, and a field of view of 152 mm x170mm, was imaged using a 3 Tesla Siemens Skyra MR Scanner with a 15 channel transmit/receive coil. Echo time was 9.4 ms and repetition time was 700 ms.

### Finite element model generation

Finite element models were established to enable systematic variation of intraoperative/ anatomical parameters that would be otherwise infeasible to evaluate experimentally for studying tissue and joint mechanics [8]. The MRI DICOM data was segmented semi-automatically using Mimics (Materialize, Belgium). Morphological noise removal and improvement of the triangulation was done according to the procedures described by Kumara [23] using Meshlab (Computing Lab-ISTI-CNR).

Simulations of different flexion-angles of the knee were performed according to Kurosawa et al. [24]. Patellar kinematics were derived from the prescribed tibiofemoral kinematics in a transient finite element simulation (ANSYS® Academic Research). The final position of the patella is determined by the forces of the quadriceps, the tension in the ligaments and the contact between patella and femur. The validity of this approach has been demonstrated by Baldwin et al. [4].

### Virtual surgery and model configuration

**Fig. 1:**
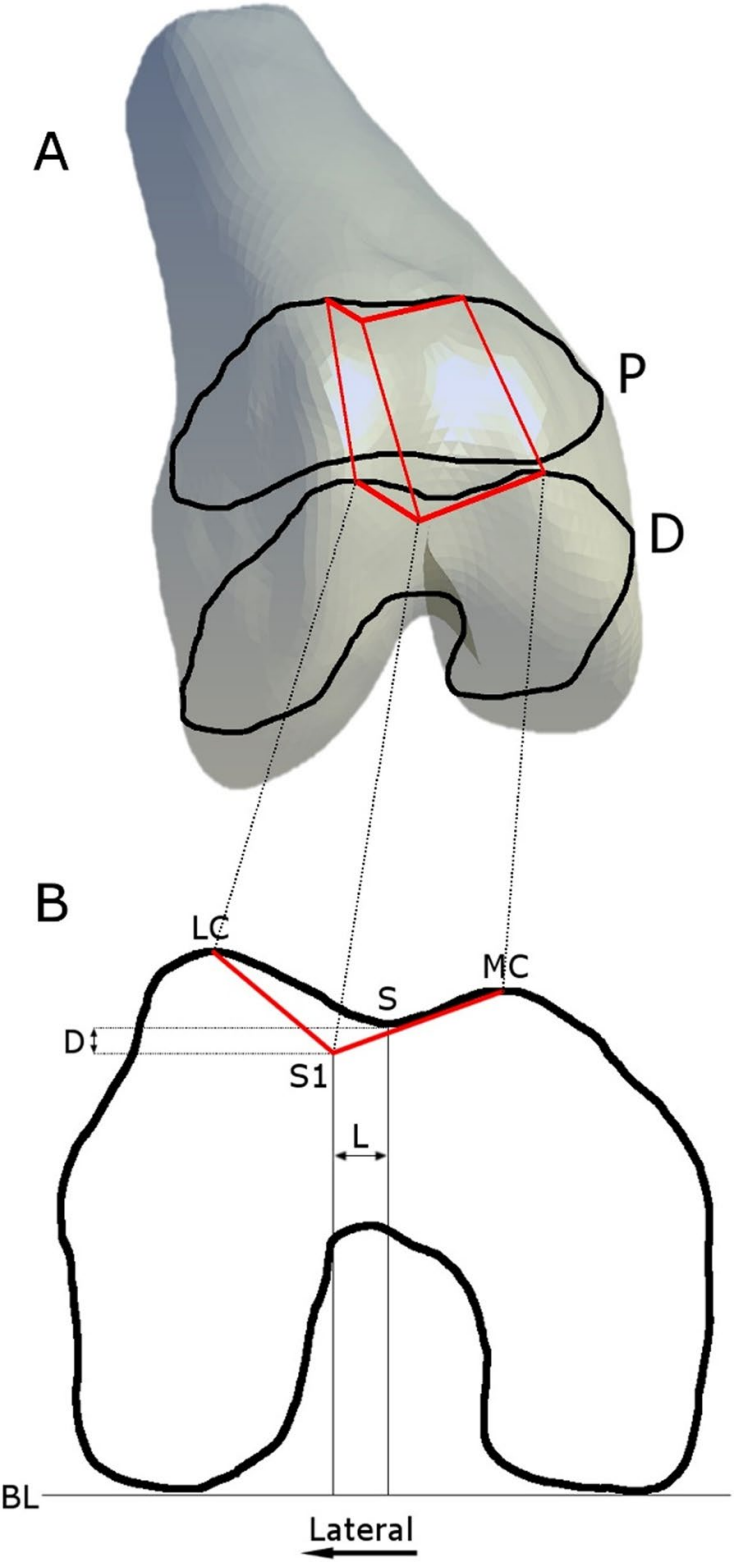
**A** Anterior view of the distal femur. Superimposed are the proximal (P) and the distal (D) schematic measurement planes used to virtually osteotomize the femur. The proximal plane is defined by the axial MR image, when the trochlea is initially completely covered with cartilage. The distal plane is defined by the axial MR image before the trochlear groove curves into the femoral notch. The wedge containing the osteotomized bone is depicted in red. **B** Schematic axial slice of the distal femur showing the virtual osteotomy conducted. The sulcus (S) is lateralized (L) and deepened (D) according to the reported results by Fucentese et al. [16], oriented parallel and perpendicular to the baseline (BL) respectively. The bone anterior to the line (depicted in red) connecting the most prominent lateral condylar point (LC), the newly defined sulcus (S1) and the most prominent medial condylar point (MC) is osteotomized

Based on the results of Fucentese et al. [16] virtual surgery for a sulcus deepening trochleoplasty [5] was performed using modeling software (Blender 2.72, Blender Foundation, Netherlands). This surgical technique consists of a lateral parapatellar approach to expose the trochlea. The cartilage along with 2 mm of bone is then separated and retracted with an osteotome. In a next step the subchondral groove is deepened and lateralized. The osteochondral flap is pressed in the newly formed bony groove and fixed with transosseous sutures. The effect of the sulcus deepening procedure is schematically shown in Fig. 1. Starting from the deepest point of the original sulcus we applied the reported mean values [16] of lateralization (L) and deepening (D) to our knee model to simulate the new sulcus (S1), this was performed on two axial images: a proximal one, when the trochlea is initially completely covered with cartilage and a distal one, before the trochlear groove curves into the femoral notch in between the values were extrapolated. To investigate the influence of over- and undercorrection on the articular cartilage pressure we arbitrarily applied the factor 1.5 and 0.5 to the reported mean values of lateralization (L) and deepening (D). Thus, virtual surgery led to a reduction of the TT-TG by 3.05 mm to 9.15 mm (0.5x mean to 1.5x mean).

Quasi-static loading simulations using a sparse matrix direct FE solver with an Augmented Lagrange contact formulation and a Gaussian contact detection (Ansys® Academic Research, Release 15.0). Tissue materials were taken from literature. Bones were treated as rigid structures, which reduced the computational time and has a negligible effect on the model predictions [20]. The articular cartilage of the patella and femur was modelled as homogeneous isotropic tetrahedral continuum elements with an elastic modulus of 5.0 MPa [36], Poisson’s ratio was 0.47 [6, 14] and a total number of deformable elements of 39,686 and 35,070 for the healthy and the trochleodysplastic knee model respectively. Cartilage at the bone-cartilage interface was rigidly fixed to the underlying bone [36]. Cartilage-cartilage friction coefficient was 0.02 [14, 36] . The patellar tendon was represented by three uniaxial spring elements [14] with a total stiffness of 4334 N/mm [19]. The femur and the tibia were fixed in space. In the quasi-static simulation, the three rotational degrees of freedom of the patella were constrained [14]. Medial- and lateral patellofemoral ligaments were modelled as uniaxial spring elements with prestrain and a stiffness of 6.45 N/mm and 5.42 N/mm respectively derived from stress-strain curves and respective cross-sectional areas [28]. The medial patellofemoral ligament was attached to the femur distally to the adductor tubercle and to the patella proximally to the midpoint of the medial patellar edge [13, 37]. Insertion points for the lateral patellofemoral ligament were set at the most lateral point of the patella and at the lateral femoral epicondyle [27]. The quadriceps muscle was modeled as three functional groups (rectus femoris/vastus intermedius, vastus medialis and vastus lateralis). The total applied muscle force was 276 N, distributed based on reported cross-sectional areas as follows: RF/VI: 111 N, VM 67 N, and VL: 98 N [32, 33, 41]. The direction of the muscular forces was adopted from Powers et al. [33] and bones were treated as rigid structures [20]. Sensitivity analyses were performed to investigate how changes in assumed cartilage material properties, thickness and FE model boundary conditions, affected predicted peak equivalent stress (Von Mises) on the patellar cartilage surface at the chondro-chondral interface [1]. This location was chosen because it showed to be least prone to artefacts, facilitation fully automated comparison. Further, peak stress predictions show to be more sensitive to changes in model assumption than average stress and contact area [1]. To account for segmentation errors [2] and to quantify the effect of necessary changes to the cartilage thickness map of the trochlear cartilage by virtually deforming it, cartilage thickness was varied by ±40%. The unfiltered cartilage surface representations were enlarged. Updated tetrahedral volumetric meshes were generated based on the surfaces. The effect of cartilage thickness was measured on the trochleodysplastic knee model postoperatively at 45° flexion. All other sensitivity studies were performed on the healthy knee model at 45° flexion.

### Model output & post processing

Articular cartilage stress was described using two scalar values: (1) contact stresses normal to the joint surface, and (2) Von Mises equivalent stress (VMes) as a general measure of the degree to which cartilage tissue is loaded. These measures reflect different aspects of the tissue level material stress fields, with contact pressure reflecting the compressive components of the stress tensor that acts on the cartilage, and VMes reflecting the shearing components of the stress tensor that tends to distort the tissue [25, 42]. To produce relevant measures of mean contact stress and VMes, only elements with stress values higher than 271 kPa were included as previously established in the literature [14]. Inclusion of all elements would lead to incomparable low stress values. Finally, contact area was computed by summing the area of each contacting patellar face.

### Statistical analysis

Statistical analysis was performed using Student’s t-test or Welch’s t-tests for unequal variances to compare the mean pressure values of the four different groups over all flexion angles. Differences were considered to be statistically significant for *p*-values < 0.05. Results are reported with mean, standard deviation and associated p-values if not stated otherwise.

## Results

### Cartilage stress

**Fig. 2:**
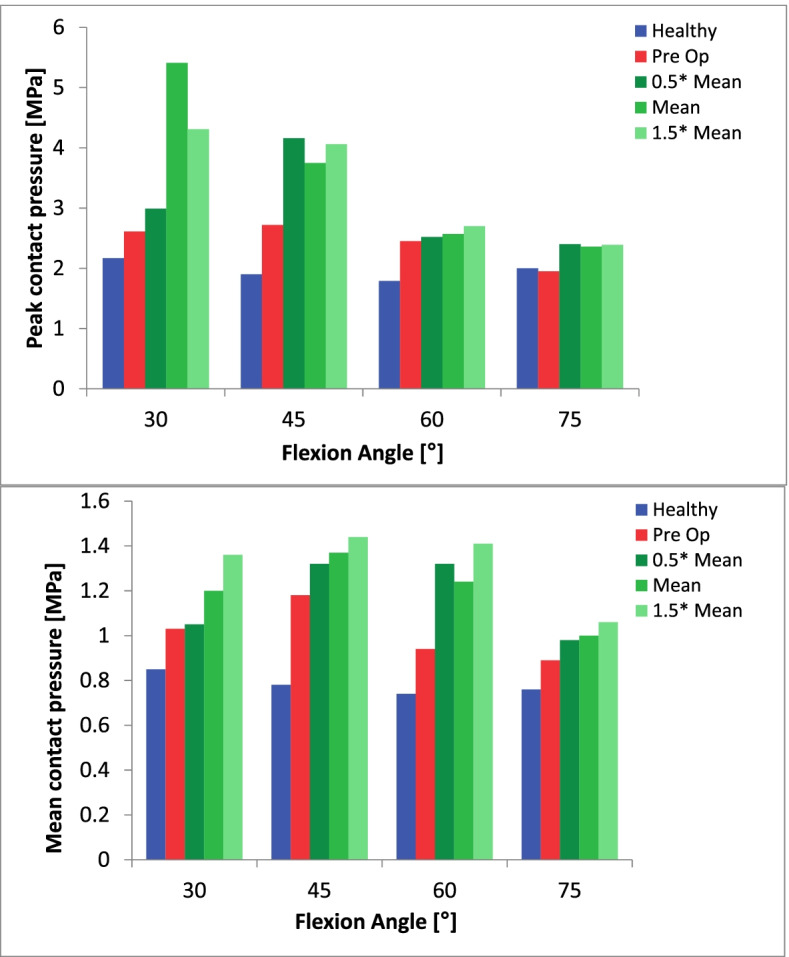
Peak and mean contact pressure subdivided into knee flexion angle for the „healthy knee“model (blue) and for the „trochleodysplastic knee“model both pre-(red) and postoperatively (green) are depicted. The latter is depicted with varied surgical parameters ranging from 0.5x mean to 1.5x mean

**Fig. 3:**
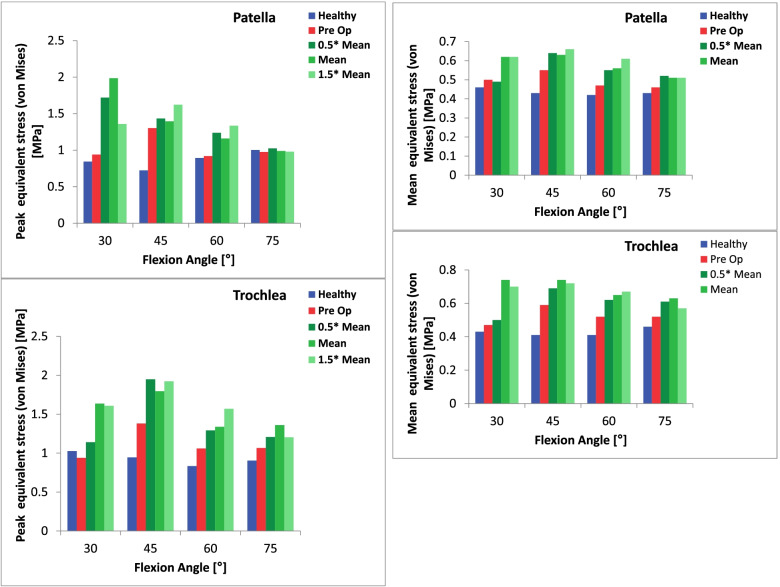
Peak (top) and mean (bottom) equivalent stress (Von Mises) at the chondro-osseous interface of the patella and trochlea subdivided into knee flexion angle for the healthy knee model (blue) and for the trochleodysplastic knee model pre- (red) and postoperatively (green) are depicted above. The latter with varied surgical parameters ranging from 0.5 x mean to 1.5 x mean

**Fig. 4:**
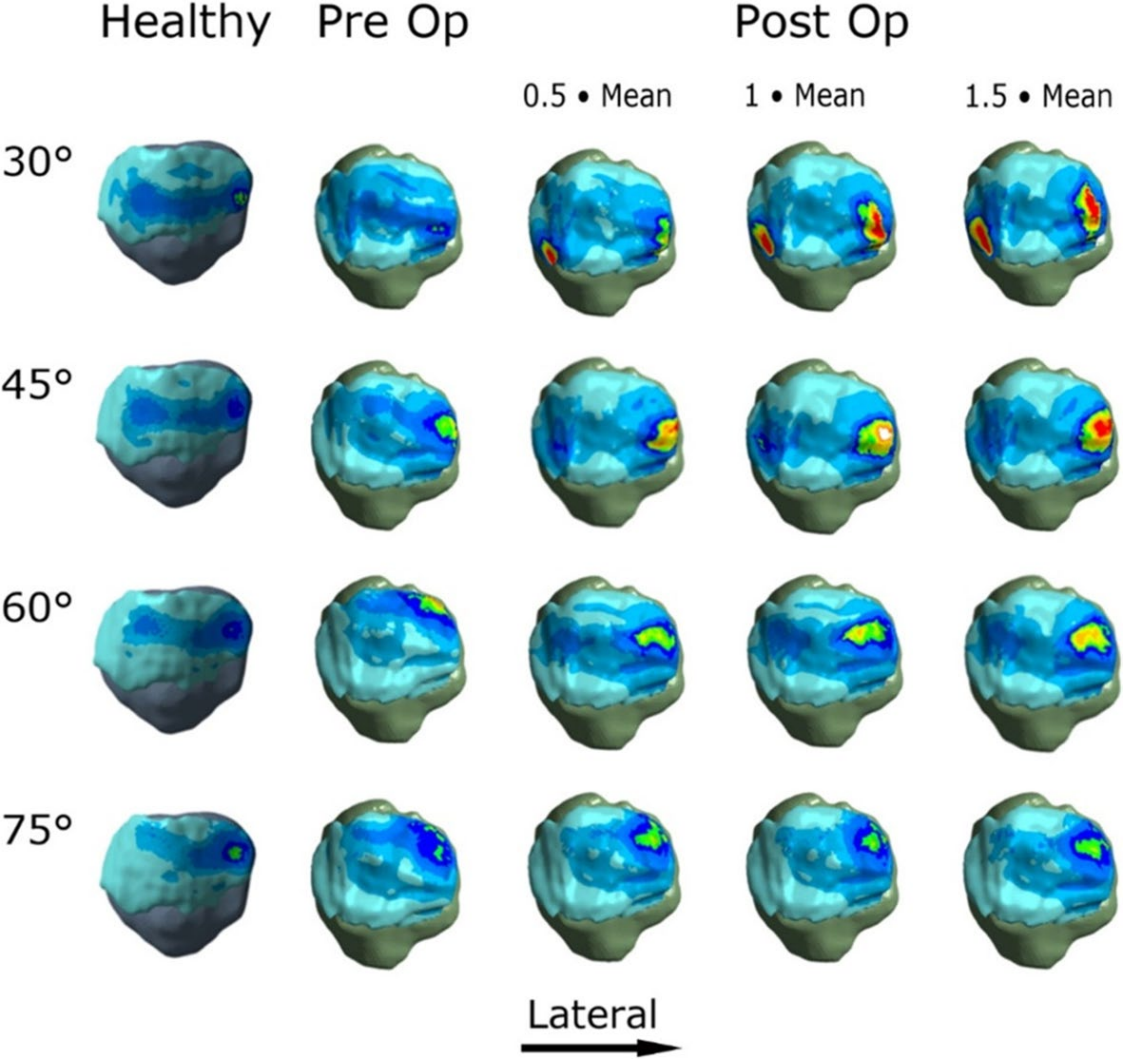
Contact pressure patterns are superimposed over the patella for the healthy control knee and the dysplastic knee pre- and postoperatively at 30°, 45°, 60° and 75° of flexion. The postoperative models show the high pressure areas marked in red. The pressure scale is constant for all models. The color assignment is nonlinear to maximize color distribution. As the healthy model represents a left knee, the respective images have been flipped to facilitate comparison. Note the pressure peaks on the lateral facet, which is pronounced at 30 to 60° flexion as well as the additional pressure peak at 30 degree flexion on the medial facet. We believe that these are evoked by the articular incongruity at the site of the greatest bony correction

**Fig. 5:**
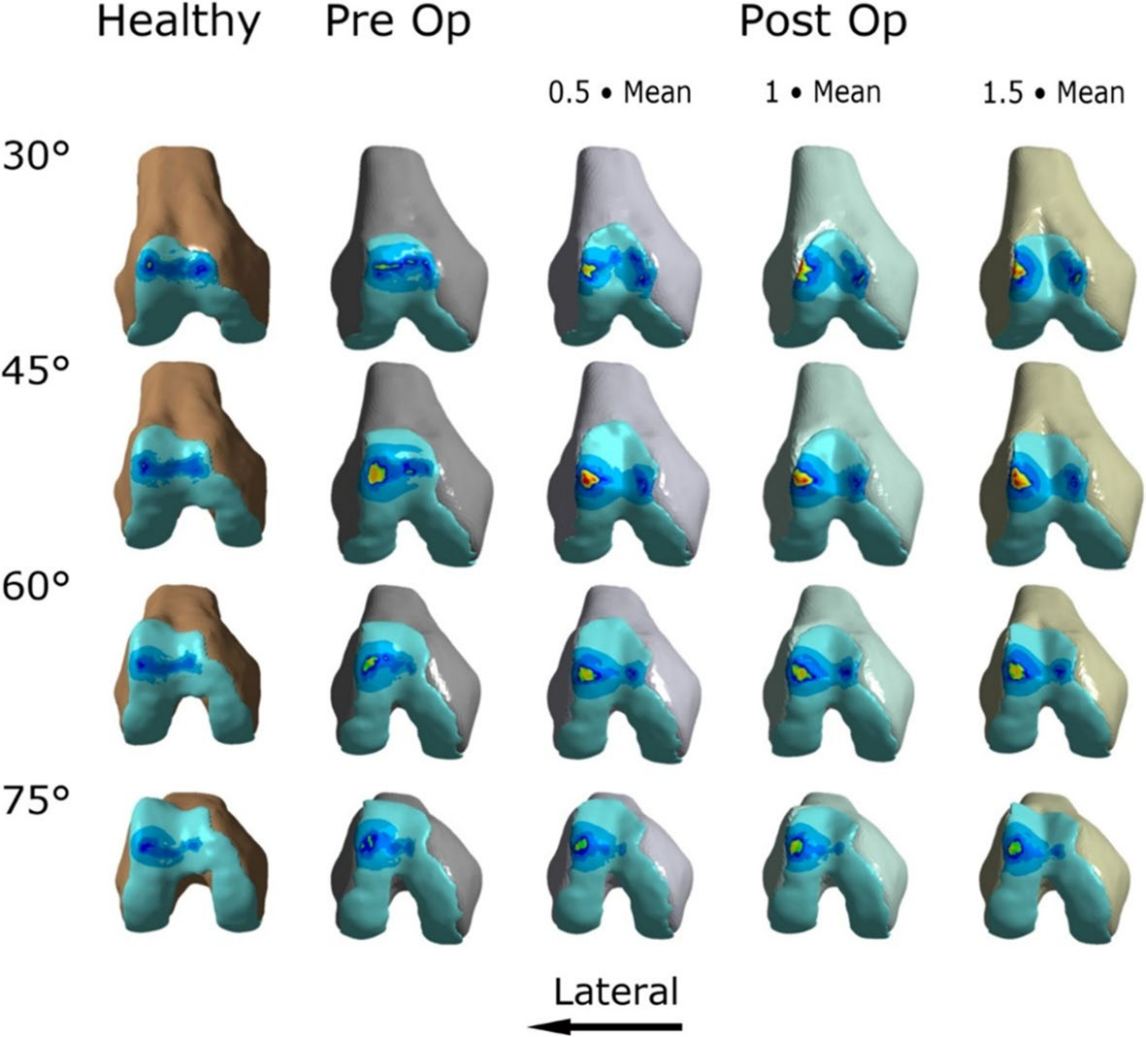
Contact pressure patterns are superimposed over the trochlea for the healthy control knee and the trochleodysplastic knee pre- and postoperatively at 30°, 45°, 60° and 75° of flexion. The pressure scale is constant for all models. The color assignment is nonlinear to maximize color distribution. As the healthy model represents a left knee, the respective images have been flipped to facilitate comparison. Note the pressure peaks on the lateral trochlea, which is pronounced at 30 to 60° flexion as well as the additional pressure peak at 30 degree flexion on the medial trochlea. We believe that these are evoked by the articular incongruity at the site of the greatest bony correction

The results regarding cartilage stress as well as the changes in patellar tilt and bisect offset are summarized in Tables 1 and 2 and Figs. 2 and 3. For both knee models, contact pressures tended to be concentrated on the lateral facet of the patella and the lateral trochlea (Figs. 4, 5).

**Table 1:** Peak and mean contact pressure between the patella and the trochlea in the healthy knee and the trochleodysplastic knee are shown “preoperative” and after simulated surgery at different flexion angles. Additionally, the patellar tilt and bisect offset values are included. * Welch’s t-test

	Healthy knee	« Preoperative »	« 0.5x mean »	« mean »	« 1.5x mean »
**Patellar tilt [°]**	12	18	9	7	5
**Bisect offset [%]**	48.5	76	65	58	53
**Flexion angle [°]**	**Peak** **[mPa]**	**Peak** **[mPa]**	**Peak** **[mPa]**	**Peak** **[mPa]**	**Peak** **[mPa]**
**30**	2.17	2.61	2.99/1.05 *+ 15%/+ 2%*	5.41 *+ 107%*	4.31 *+ 65%*
**45**	1.9	2.72	4.16/1.32 *+ 52%/+ 12%*	3.75 *+ 38%*	4.06 *+ 49%*
**60**	1.79	2.45	2.52/1.32 *+ 3%/+ 40%*	2.57 *+ 5%*	2.7 *+ 10%*
**75**	2	1.95	2.4/ 0.98 *+ 23%/+ 10%*	2.36 *+ 21%*	2.39 *+ 23%*
**All angles**	***1.97 (SD 0.16)***	***2.43 (SD 0.34)***	***3.3 (SD 1.01)***		
**Significance**		*P* = 0.047* vs. healthy	*P* = 0.022* vs. preoperative		
**Flexion angle [°]**	**Mean** **[mPa]**	**Mean** **[mPa]**	**Mean** **[mPa]**	**Mean** **[mPa]**	**Mean** **[mPa]**
**30**	0.85	1.03	1.05 *+ 2%*	1.2 *+ 17%*	1.36 *+ 32%*
**45**	0.78	1.18	1.32 *+ 12%*	1.37 *+ 16%*	1.44 *+ 22%*
**60**	0.74	0.94	1.32 *+ 40%*	1.24 *+ 32%*	1.41 *+ 50%*
**75**	0.76	0.89	0.98 *+ 10%*	1 *+ 12%*	1.06 *+ 19%*
**All angles**	***0.79 (SD 0.05)***	***1.01 (SD 0.13)***	***1.23 (SD 0.17)***		
**Significance**		*P* = 0.016* vs. healthy	*P* = 0.033* vs. preoperative

**Table 2:** Peak and mean equivalent stress (Von Mises) at the patellar and the trochlear cartilage are shown in the healthy knee and the trochleodysplastic knee “preoperative” and after simulated surgery at different flexion angles. “Welch’s t-test.

	Healthy knee	« Preoperative »	« 0.5x mean »	« mean »	« 1.5x mean »
**Flexion angle [°]**	**Peak Patella /Trochlea** **[mPa]**	**Peak Patella /Trochlea** **[mPa]**	**Peak Patella /Trochlea** **[mPa]**	**Peak Patella /Trochlea** **[mPa]**	**Peak Patella /Trochlea** **[mPa]**
**30**	0.844/ 1.027	0.94/ 0.939	1.719/ 1.141 *+ 82%/ + 22%*	1.985/ 1.636 *+ 111%/ + 74%*	1.359/ 1.608 *+ 45%/ + 71%*
**45**	0.724/ 0.945	1.302/ 1.381	1.433/ 1.948 *+ 10%/* 41%	1.395/ 1.795 *+ 7%/ + 30%*	1.623/1.923 *+ 25%/ + 39%*
**60**	0.893/ 0.834	0.919/ 1.06	1.238/ 1.293 *+ 35%/ + 22%*	1.16/ 1.338 *+ 26%/ + 26%*	1.336/ 1.57 *+ 45%/ + 48%*
**75**	1.002/ 0.905	0.975/ 1.067	1.025/ 1.208 *+ 5%/ + 13%*	0.989/ 1.36 *+ 1%/ + 27%*	0.979/ 1.204 *+ 0%/ + 13%*
**All angles**	***0.87 (SD 0.12)/ 0.93 (SD 0.08)***	***1.03 (SD 0.18)/ 1.11 (SD 0.19)***	***1.35 (SD 0.31)/ 1.5 (SD 0.28)***		
**Significance**		*P* = 0.167*/ *P* = 0.123* vs. healthy	*P* = 0.073*/ *P* = 0.024* vs. preoperative		
**Flexion angle [°]**	**Mean Patella /Trochlea** **[mPa]**	**Mean Patella /Trochlea** **[mPa]**	**Mean Patella /Trochlea** **[mPa]**	**Mean Patella /Trochlea** **[mPa]**	**Mean Patella /Trochlea** **[mPa]**
**30**	0.46/ 0.43	0.5/ 0.47	*0.49/ 0.5* *−2%/ + 6%*	0.62/ 0.74) + 24%/ + 57%	*0.62/ 0.7* *+ 24%/ + 49%*
**45**	0.43/ 0.41	0.55/ 0.59	0.64/ 0.69 + 16%/ + 16%	0.63/ 0.74 + 15%/ + 25%	0.66/ 0.72 + 20%/ + 22%
**60**	0.42/ 0.41	0.47/ 0.52	*0.55/ 0.62* *+ 17%/ + 19%*	*0.56/ 0.65* *+ 19%/ + 25%*	*0.61/ 0.67* *+ 30%/ + 29%*
**75**	0.43/ 0.46	0.46/ 0.52	0.52/ 0.61 + 13%/ + 17%	*0.51/ 0.63* *+ 10%/ + 21%*	*0.51/ 0.57* *+ 10%/ + 9%*
**All angles**	***0.43 (SD 0.02)/ 0.43 (SD 0.02)***	***0.49 (SD 0.04)/ 0.52 (SD 0.05)***	***0.58 (SD 0.06) / 0.65 (SD 0.07)***		
**Significance**		*P* = 0.041*/ *P* = 0.01* vs. healthy	*P* = 0.02*/ *P* = 0.006* vs. preoperative

### Contact area

Over the four assed knee flexion angles the patellofemoral contact area between the “healthy” and the “pathological” knee model did not differ significantly (640 mm^2^ (SD 96.4 mm^2^) vs 598.1 mm^2^ (SD 37.6mm^2^, *P = 0.449*).

**Fig. 6:**
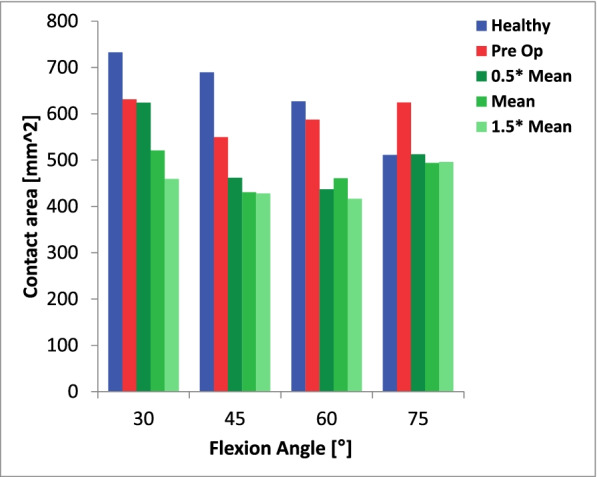
Patellofemoral contact area as a function of knee flexion angle for the healthy knee model (blue) and for the trochleodysplastic knee both pre- (red) and postoperatively (green) are depicted above. The latter with a varied cut size from 0.5x mean to 1.5x mean

Virtual deepening trochleoplasty significantly decreased patellofemoral contact area over all assessed knee flexion angles (598.1 mm^2^ (SD 37.6 mm^2^) vs 478.4 mm^2^ (SD 57.2mm^2^), *P = 0.002*) (Fig. 6).

## Discussion

Our main objective was to establish an FE model to quantitatively investigate the effect of sulcus deepening trochleoplasty on retropatellar contact pressure distribution. We specifically aimed to assess the plausibility that accelerated retropatellar and trochlear degeneration as well as anterior knee pain could be driven by joint surface anatomy in cases of severe trochlear dysplasia, and further that corrective surgery may exacerbate joint tissue loads.

In our model we noted a significantly greater mean and peak contact pressure (*P* = 0.016 and *P* = 0.047, respectively) in the simulated trochleodysplastic knee for all investigated flexion angles with equal loading protocols (Fig. 2). A further significant increase after simulated sulcus deepening surgery (*P* = 0.033 and *P* = 0.022, respectively) was noted. We judge it to be unlikely that these differences are solely due to natural anatomical variance, as experimental studies using pressure sensitive films have reported smaller relative deviations with confidence intervals of 10–14% [21, 33]. Possible explanations for this observation may be the smaller contact area in our trochleodysplastic knee model, which further decreases significantly after virtual deepening trochleoplasty (*P* = 0.002) (Fig. 6). Another explanation may be the geometrical incongruity between the patella and the trochlea and the anti Maquet effect preoperatively (Fig. 4) [15].. After virtual deepening trochleoplasty the contact pressure further increases significantly exceeding a potential Maquet effect in all simulations .

Calculations of mean and peak VMes values were additionally performed in our model, as increased shear stress may negatively influence chondrocyte metabolism and accelerate progression of osteoarthritis [25, 26, 40, 42]. Shear stress cannot be measured directly, and as such are not commonly used in literature, making comparison difficult. Mean VMes were predicted to increase significantly both on the patella and the trochlea with peak VMes predicted to be significantly raised at the surgically corrected trochlea. Again, we attribute this finding to the increased geometrical incongruity between the unaltered patella and the deepened sulcus.

The link between trochlear dysplasia and patellofemoral arthritis is well established [9, 17, 22]; with specific correlation between arthritis and the various forms of dysplasia [9]. After surgical correction of patellar instability by trochleoplasty in trochlear dysplasia progression of patellofemoral osteoarthritis remains a relevant clinical concern [39] despite promising mid-term clinical results [32].

With this model we have raised a possible theory to help explain these clinical observations.

To what extent trochleoplasty surgery influences further cartilage breakdown is unknown; however, the present work shows that in our model significantly higher cartilage loads occur in a trochleodysplastic knee joint compared to a healthy knee joint under the same loading condition. Simulation of sulcus deepening surgery further significantly increased cartilage load.

The greatest limitation of this pilot study is that only one healthy and one “trochleodysplastic” knee were developed and investigated. It would be premature to draw direct conclusions from these results for everyday clinical practice and more knee joints must be included to increase validity of our finding. Further limitations include simplifications on patellofemoral kinematics by using values reported in literature [29, 33] and simplifications of the FE model (linear elastic cartilage and rigid bone). Another limitation includes the low muscle forces compared to daily activities such as walking and running. The low muscle force was deliberately chosen to indirectly validate the study to the literature [32].

**Fig. 7:**
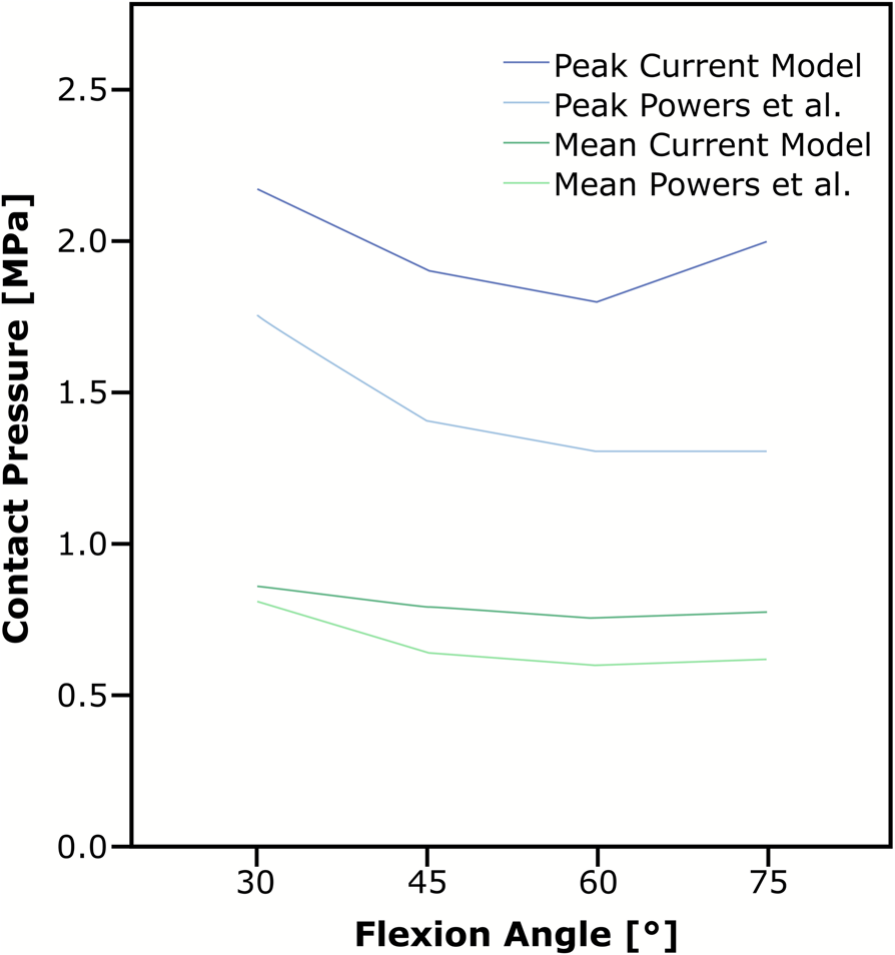
Peak and mean contact pressure as a function of knee flexion angle are illustrated for both the healthy knee model (FE calculation) and values reported by Powers et al. [33]. The “healthy knee” model in the current study consistently predicted significantly higher peak pressure values (1.97 MPa (SD 0.16 MPa) vs. 1.44 MPa (SD 0.21 MPa), *p = 0.007*) and slightly higher mean pressure values (0.79 MPa (SD 0.05 MPa) vs. 0.66 MPa (SD 0.02 MPa), *p = 0.054*)

Despite these limitations, both models consisted with experimental data that support their validity. An indirect validation was performed by comparing our predicted results to values reported in the literature [33]. The same loading protocol was applied. The “healthy” knee model in our study consistently predicted slightly higher mean pressure values (0.79 MPa (SD 0.05 MPa) vs. 0.66 MPa (SD 0.02 MPa), *P = 0.054*) and significantly higher peak pressure values (1.97 MPa (SD 0.16 MPa) vs. 1.44 MPa (SD 0.21 MPa), *P = 0.007*) as seen in Fig. 7. As the read- out percentage differs from the method used in the literature [33] no statement can be made regarding peak contact pressures. Mean pressure values were slightly, but consistently higher, but nonetheless within the approximate 10% margin of error for pressure sensitive films used in such pressure measurements [18]. Mean contact area was markedly higher at all flexion angles [33], however we attribute this to the relative lack of sensitivity of the pressure sensitive films used in the experimental studies (0.33 MPa- 2.94 MPa) [32, 38] – an example of the limitation of experimental methods that computational models can be used to overcome.

## Conclusions

In this pilot study our model predicts that patellofemoral contact pressure and shear stress is significantly higher in a trochleodysplastic knee compared to a healthy knee and further significantly increases after sulcus deepening surgery. Further studies are necessary to investigate whether this finding has a decisive influence on early patellofemoral osteoarthritis in this patient collective as observed in clinical practice.

## Data Availability

The datasets used and/or analysed during the current study are available from the corresponding author on reasonable request.

## Abbreviations

FE: Finite element
LPFL: Lateral patellofemoral ligament
MPFL: Medial patellofemoral ligament
PD: Proton density
SPACE: Sampling perfection with application-optimized contrasts using different flip angle evolutions
VMes: Von Mises equivalent stress
